# Comprehensive Pan-Cancer Analysis of GINS2 for Human Tumour Prognosis and as an Immunological Biomarker

**DOI:** 10.1155/2022/3119721

**Published:** 2022-11-23

**Authors:** Wei Meng, Zhaosheng Jiang, Xiang Zhang, Bo Cai, Limin Ma, Yangbo Guan

**Affiliations:** Department of Urology, Affiliated Hospital of Nantong University, Medical School of Nantong University, Nantong 226001, China

## Abstract

**Background:**

In recent years, more and more reports have shown that GINS complex subunit 2 (GINS2) plays an important role in the occurrence and progression of tumours. However, there is a lack of comprehensive and systematic research on its prognostic and immune effects in pan-cancer. Therefore, this study is aimed at investigating the prognostic value and immune-related role of GINS2 in human tumours and providing a comprehensive understanding of its carcinogenic mechanism in pan-cancer.

**Methods:**

We investigated different databases, including TIMER, TCGA, GTEX, CPTAC, GEPIA, and SangerBox. The study was carried out on the expression and prognosis of GINS2 in human tumours, immune infiltration and microenvironment, immune checkpoints, neoantigens, tumour mutational burden, microsatellite instability, mismatch repair (MMR) genes, methylation, cancer-associated fibroblasts (CAFs), and enrichment analysis of gene set.

**Results:**

GINS2 plays a potential carcinogenic role in various human tumours through mRNA and protein levels. It is highly expressed in most cancers, and its expression is significantly correlated with tumour prognosis. In addition, the expression of GINS2 is associated with immune microenvironment and immune infiltration, especially in brain lower-grade glioma, lung squamous cell carcinoma, TGCT, breast invasive carcinoma, and glioblastoma multiforme. At the same time, GINS2 is related to immune neoantigens and the expression profiles of immune checkpoint genes in pan-cancer. It also affects the expression of DNA MMR genes and methyltransferase in pan-cancer. Finally, the correlation between GINS2 and CAF abundance in most tumours was studied, and an enrichment analysis of GINS2 and its related proteins was also carried out.

**Conclusion:**

This is the first study on GINS2 as a prognostic and immune mechanism in pan-cancer. GINS2 may be a valuable prognostic immunological biomarker of pan-cancer. This paper provides a relatively comprehensive understanding on the correlation of GINS2 with pan-cancer.

## 1. Introduction

Cancer is the leading cause of death in countries around the world. According to the latest statistics, the incidence and mortality of cancer are very high and have become a severe public health problem [1]. Because the mechanisms of tumourigenesis and progression are very complex and specific, the interaction between tumour and immunity remains a hot research topic [2]. In recent years, the tumour microenvironment has been believed to play an essential role in tumour progression, and conversely, tumour progression can lead to immune suppression of the tumour microenvironment [3]. As an essential component of the tumour microenvironment, immune cells are involved in immune regulation and play a key role in cancer progression [4]. In addition, many novel immune checkpoints are gradually being discovered [5, 6]. In conclusion, there is growing evidence that immune-related mechanisms play an essential role in human tumourigenesis and progression [7]. Therefore, it has become very urgent to study the mechanisms of human tumourigenesis and progression and seek immune biomarkers that can assist in diagnosis, prognosis, and management [8]. With the continuous improvement of data from the Cancer Genome Atlas (TCGA), Genotype-Tissue Expression (GTEx), Clinical Proteomic Tumor Analysis Consortium (CPTAC), and other databases, we can explore the correlation between the expression of selected genes and the clinical prognosis, immunity, and various pathways of pan-cancer by performing pan-cancer analysis [9] and thus can better understand the relationship between immune mechanisms tumourigenesis and progression.

As one of the GINS family, GINS complex subunit 2 (GINS2, molecular weight of 1196 bp mRNA and~21 kDa) is a gene with a replicative helicase structure, which exists in human chromosome 16q24. It plays a vital role in initiating DNA replication and cellular processes. The expression of the GINS2 gene is upregulated significantly in cholangiocarcinoma and stage II lung adenocarcinoma. The downregulation of GINS2 expression can inhibit the growth of breast cancer cells by activating endogenous DNA damage [10–12]. Some reports show that GINS2 is also involved in the occurrence and progression of various cancers [13], including breast cancer, ovarian cancer, cervical cancer, leukemia, and glioma [14]. Therefore, we can understand the critical role of the GINS2 gene in cancers. Unfortunately, no comprehensive analysis on the role of GINS2 in pan-cancer has been reported.

**Figure 1:**
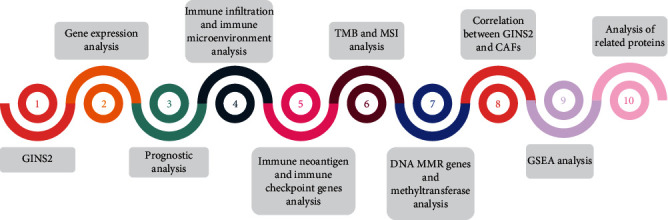
A framework that summarizes the whole research content.

This study investigated the Tumor IMmune Estimation Resource (TIMER), TCGA, GTEX, CPTAC, GEPIA, and other databases. We performed a relatively comprehensive bioinformatic analysis on the clinical prognosis and immunity of GINS2 in pan-cancer (as shown in Figure 1). The results showed that GINS2 might be a valuable prognostic immunological biomarker of pan-cancer.

## 2. Materials and Methods

### 2.1. Gene Expression Analysis

Firstly, the expression differences of GINS2 in different tumours and adjacent tissues from the TCGA database were explored in the “Gene_DE” module in TIMER2.0 (Tumor IMmune Estimation Resource, version 2) (http://timer.cistrome.org/) [15]. To ensure the relative comprehensiveness of the study, tumours that cannot be used to make matched-pair analysis with normal tissues in the TCGA database were analyzed supplementarily in the GTEx (Genotype-Tissue Expression) database based on the GEPIA2 (Gene Expression Profiling Interactive Analysis, version2) [16] website (http://gepia2.cancer-pku.cn/#analysis->BoxPlot). The following parameters were set: *P* value cutoff = 0.01 and log2FC (fold change) cutoff = 1.

The interactive network resource UALCAN portal (http://ualcan.path.uab.edu/analysis-prot.html) was used to study the expression differences of GINS2 protein in tumour tissues of different tumours. The “GINS2” gene was input in the “CPTAC analysis” module. It was normalized to the standard deviation of the median by using the expression value recorded by each proteomic spectrum in the CPTAC database to compare the protein expression of GINS2 between tumour and normal tissues. We selected the available data sets of eight tumours: glioblastoma multiforme (GBM), head and neck squamous cell carcinoma, hepatocellular carcinoma, lung adenocarcinoma, ovarian cancer, pancreatic cancer, endometrial cancer, and breast cancer [17]. In addition, we studied the expression of GINS2 in different cancers and pathological stages using the “Pathological Stage Plot” module in GEPIA2 (http://gepia2.cancer-pku.cSurvivalAnalysis) and drew the violin plot .

Finally, using cBioPortal (http://www.cbioportal.org/), we studied the genomic changes of GINS2 in different cancers. We presented the genome changes of GINS2 in the TCGA pan-cancer database (mutation, structural variant, amplification, and deep deletion) in the form of images to make them visible .

### 2.2. COX Regression Analysis and Survival Analysis

Considering that there are other causes leading to the death of patients in addition to tumour causes in the follow-up examination, patients were divided into two groups in SangerBox (http://www.sangerbox.com/tool), namely, high expression and low expression, based on the median of GINS2 expression level to analyze the correlation between GINS2 expression and prognosis, including overall survival (OS), disease-specific survival (DSS), disease-free interval (DFI), and progression-free interval (PFI). Single-factor analysis was used to calculate the risk ratio and 95% confidence interval. The results are shown in the Kaplan–Meier curves and forest plot.

### 2.3. Correlation Analysis between Immune Infiltration and Microenvironment

Some reports show that tumour-infiltrating lymphocytes are in the sentinel lymph node state of cancer and play an essential role in predicting survival in cancer. Therefore, we studied the relationship between the expression of GINS2 and immune cells using the TIMER database and presented the immune cell scores of different tumour samples through immune scoring, interstitial scoring, and ESTIMATE score (setting parameter *P* < 0.05 and *R* > 0.20) [18]. High ImmuneScore and StromalScore are positively correlated with immune score and substrate ratio. The ESTIMATEScore is a composite score representing the combined ratio of two components in TME.

### 2.4. Correlation Analysis between Immune Checkpoints and Immune Neoantigen

In recent years, immune checkpoint blockade therapy has shown significant efficacy in treating various tumours. It can inhibit the binding of programmed death receptors and their ligands to improve the aggression of the host immune system against tumour cells. To study the relationship between GINS2 gene expression and immune checkpoint gene expression, more than 40 common immune checkpoint genes were included in calculating their correlation with GINS2. Tumour neoantigen is a tumour-specific antigen produced by the expression of nonsynonymous mutations due to the genetic instability of tumour cells, which often leads to many mutations. Some neoantigens can be expressed, processed, and presented on the cell surface and then recognized by T cells under the molecules of major histocompatibility complexes.

At present, tumour neoantigen has become an ideal target for T cell-based cancer immunotherapy. In this study, the number of tumour neoantigens of different tumour samples was accounted for, and the relationship between GINS2 gene expression and the number of tumour neoantigens was analyzed. The above two correlation analyses were carried out through the SangerBox online platform.

### 2.5. Correlation Analysis between GINS2 and TMB and Microsatellite Instability (MSI)

The tumour mutational burden (TMB) refers to the total number of somatic variations detected per million primary groups. The results showed that patients with high TMB (TMB-H) are more likely to benefit from the treatment of immune checkpoint inhibitors. Therefore, TMB is becoming a new biomarker for immunotherapy of various cancers. MSI refers to the phenomenon wherein, compared with normal tissues, new microsatellite alleles appear at a microsatellite site in tumours due to the insertion or deletion of repeat units. MSI is caused by functional defects due to DNA mismatch repair (MMR) in tumour tissue. Its role in tumour immunotherapy has also been widely studied. This study analyzed the correlation between TMB-MRI and ESTIMATEScore using the SangerBox online platform.

### 2.6. DNA MMR Genes and Methylation Correlation Analysis

MMR refers to a repair method that makes the nucleotide sequence return to normal in DNA molecules containing mismatched bases. When germline mutation or methylation occurs in MMR genes, the function of MMR will decline. As a result, the DNA sequence's base mismatch, deletion, or insertion cannot be repaired. The MMR system can involve multiple MMR genes when participating in DNA repair, including two families, namely, MutS (MSH2, MSH3, and MSH6) and MutL (MLH1, MLH3, PMS1, and PMS2). Amongst them, MLH1, MSH2, MSH6, and PMS2 are the significant genes of MMR. Therefore, five MMR genes (MLH1, MSH2, MSH6, PMS2, and EPCAM) were selected, and their correlation with GINS2 expression was studied based on the TCGA database. DNA methylation is a methyl group bound by cytosine five-carbon covalent bonds of CpG dinucleotides in the gene group under the effect of DNA methylation transferase. It can change the genetic performance without changing the DNA sequence. Then, we studied the correlation between the expression of the GINS2 gene and four methyltransferases. The above study was carried out by the ggplot method (setting parameter *P* < 0.05 and *R* > 0.20).

### 2.7. Correlation Analysis between GINS2 and Cancer-Associated Fibroblasts (CAFs)

Many CAFs in tumour tissues can construct a suitable environment for tumour progression. CAFs can not only inhibit the function of immune cells through secreting a variety of cytokines or metabolites to promote the development, invasion, and metastasis of tumours. They also shape the extracellular matrix of tumours and form the permeability barrier of drugs or therapeutic immune cells to prevent their deep penetration into tumour tissue, reducing the therapeutic effect on tumours. Therefore, inhibiting tumours by regulating CAFs or overcoming their barrier effect has become a new method of tumour treatment. Four algorithms (XCELL, MCPCOUNTER, EPIC, and TIDE) were adopted in this study. The *P* value and partial correlation (cor) value were derived from the Spearman rank correlation test after purity correction. The results are presented in the form of a heat map and scatter diagram.

### 2.8. Enrichment Analysis of Gene Sets

To study the biological signal pathways of GINS2 involved in the high- and low-expression groups, gene set enrichment analysis was carried out. The results show only the first three KEGG pathways and the first four pathways of HALLMARK analysis. The net enrichment score (NES), gene ratio, and *P* value were used to screen the enrichment results of the KEGG pathway. Here, the parameters with significant effects were set as ∣NES | >1, NOM *P* < 0.05, and FDR *q* < 0.05.

### 2.9. GINS2-Associated Gene Analysis

Firstly, a signal gene name “GINS2” was input in STRING (https://string-db.org/). The organism was set as Homo sapiens. In the pop-up page, the following parameters were set: minimum required interaction score (“low confidence (0.400)”), the meaning of network edges (“evidence”), max number of interactors to show (“no more than 50 interactors”), and active interaction sources (“experiments”). Finally, 22 genes closely related to GINS2 were obtained. At the same time, based on the “Similar Gene Detection” module of the GEPIA2 (Gene Expression Profiling Interactive Analysis, version 2) platform, 100 targeted genes with the highest similarity to GINS2 were obtained from tumour and normal tissues in the TCGA database. The cross-analysis was carried out on genes interacting with GINS2 using jvenn (an interactive Venn diagram viewer). Then, the pairwise Pearson correlation analysis between the selected gene and GINS2 was carried out using the “correlation analysis” module in the GEPIA2 platform. The results were presented in the form of a scattered diagram. In addition, a heat map was provided for the selected gene using the “Gene_Corr” module of TIMER2. After purity adjustment, the data include partial correlation (cor) and *P* value in the Spearman rank correlation test.

## 3. Result

### 3.1. Gene Expression Analysis

**Figure 2:**
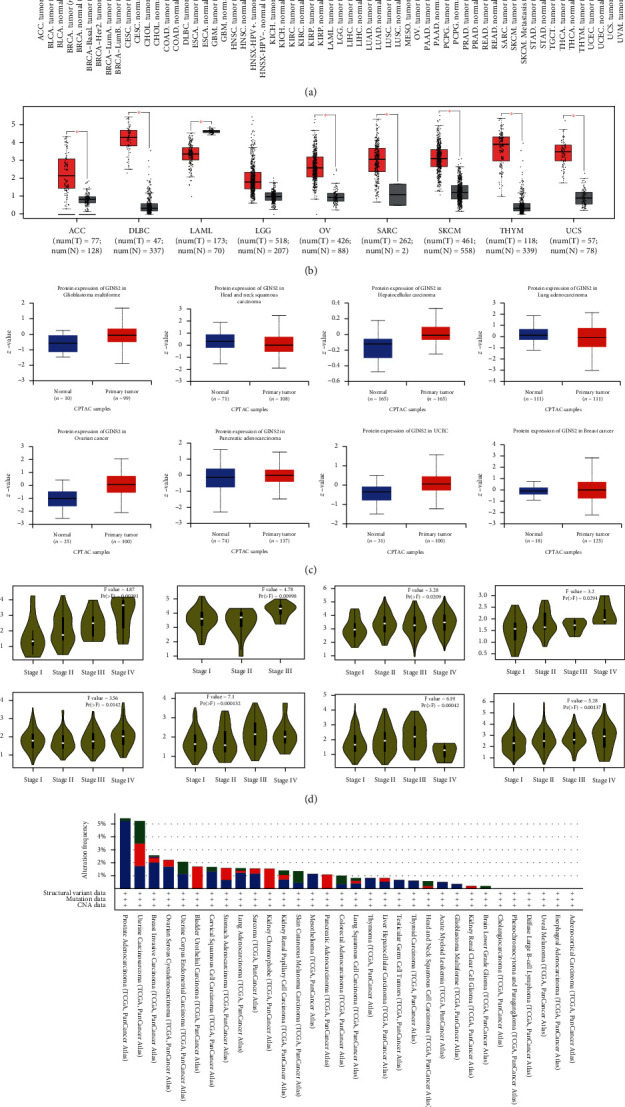
Expression of the GINS2 gene in different cancer types and pathological tumour stages. (a) Analysis of GINS2 mRNA expression in different tumours by TIMER2. ^∗^*P* < 0.05; ^∗∗^*P* < 0.01; ^∗∗∗^*P* < 0.001. (b) Differential mRNA expression of GINS2 in ACC, DLBC, LAML, OV, SARC, SKCM, THYM, and UCS versus the corresponding nontumour tissues in TCGA dataset. The box chart data are provided. ^∗^*P* < 0.05. (c) The protein expression levels of GINS2 were analyzed in tumour and paracancerous tissues of several cancers (BRCA, GBM, hepatocellular carcinoma, HNSC, LUAD, OV, PAAD, and UCEC) by the CPTAC dataset. (d) In addition, the differences in GINS2 expression levels were analyzed in the pathological stages of different tumours (ACC, HNSC, KICH, KIRC, KIRP, LIHC, LUAD, and TGCT). Log2 (TPM+1) was used for logarithmic analysis. (e) GINS2 mutation frequency in human cancers according to data on the cBioPortal database.

Firstly, the expression differences of GINS2 between cancer and normal tissue in the TCGA database by using the TIMER method are shown in Figure 2(a). The expression of GINS2 in bladder urothelial carcinoma (BLCA), breast invasive carcinoma (BRCA), cholangiocarcinoma (CHOL), colon adenocarcinoma (COAD), oesophageal carcinoma (ESCA), GBM, head, and neck squamous cell carcinoma (HNSC), kidney chromophobe (KICH), kidney renal clear cell carcinoma (KIRC), kidney renal papillary cell carcinoma (KIRP), liver hepatocellular carcinoma (LIHC), lung adenocarcinoma (LUAD), lung squamous cell carcinoma (LUSC), rectum adenocarcinoma (STAD), thyroid carcinoma (THCA), uterine corpus endometrial carcinoma (UCEC) (*P* < 0.001), cervical squamous cell carcinoma, and endocervical adenocarcinoma (CESC), rectum adenocarcinoma (READ) (*P* < 0.01), and pheochromocytoma and paraganglioma (PCPG) (*P* < 0.05) is significantly higher than that in normal tissue. Then, the differences in GINS2 expression in adrenocortical carcinoma (ACC), lymphoid neoplasm diffuse large B cell lymphoma (DLBC), acute myeloid leukemia (LAML), brain lower grade glioma (LGG), ovarian serous cystadenocarcinoma (OV), sarcoma (SARC), cutaneous skin melanoma (SKCM), thymoma (THYM), and uterine carcinosarcoma (UCS) from that in normal tissue were evaluated. The results showed that the expression of GINS2 in ACC, DLBC, LAML, OV, SARC, SKCM, THYM, and UCS (*P* < 0.05) was significantly higher than that in normal tissue (Figure 2(b)).

In terms of protein level, based on the analysis result of the CPTAC data set, the total GINS2 protein expression is higher in GBM, hepatocellular carcinoma, OV, PAAD, UCEC, and BRCA (*P* < 0.001). In contrast, the expression in HNSC and LUAD is lower than that in normal tissue (Figure 2(c)). Whether GINS2 genes are different in different pathological stages of other cancers has also been studied. The result showed that the expression of GINS2 is significantly correlated with ACC (*P* = 0.00391), HNSC (*P* = 0.0209), KICH (*P* = 0.0294), KIRC (*P* = 0.0142), KIRP (*P* = 0.000132), LIHC (*P* = 0.00042), LUAD (*P* = 0.00137), and TGCT (*P* = 0.00998) in different pathological stages (Figure 2(d)). With the gradual increase of the pathological stages of ACC, HNSC, KIRC, KIRP, LUAD, and TGCT tumours, the expression of GINS2 also increased accordingly. Therefore, the expression of GINS2 can promote the progression of these tumours. Finally, the changes in the GINS2 gene in 33 tumours and 10,953 patients were studied based on the cBioPortal network platform. The results showed that GINS2 gene changes occurred in 154 (1.4%) of 10,953 patients. The first three genes with the highest GINS2 gene change rate occurred in prostate cancer (5.47% in 494 cases), uterine sarcoma (5.26% in 57 points), and invasive breast carcinoma (2.58% in 1084 cases) (Figure 2(e)).

### 3.2. Prognostic Analysis of GINS2 in Pan-Cancer

**Figure 3:**
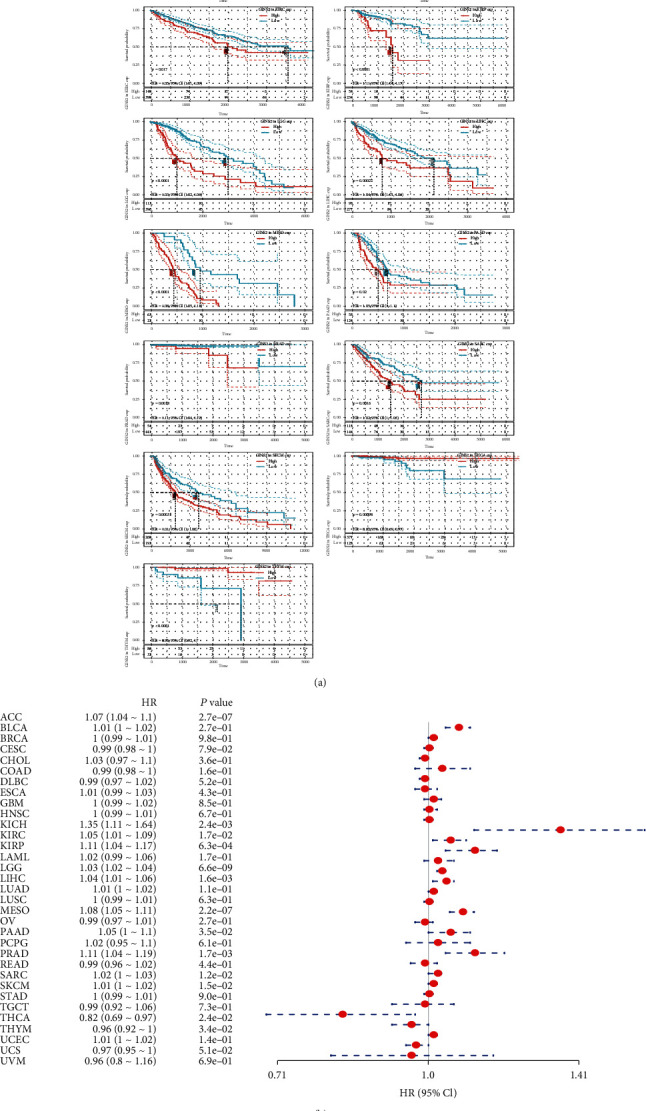
Correlation between GINS2 gene expression and survival prognosis of cancers in TCGA. The Kaplan–Meier curves of OS in different tumours with positive results are offered.

Firstly, the correlation of GINS2 expression in 33 tumours with OS, DSS, DFI, and PFI was studied using single-factor Cox analysis based on TCGA database. The results are presented in the form of a forest map and Kaplan–Meier curve (Figures 3(a) and 3(b) and Figures S1, S2, and S3). From the analysis results, the high expression of GINS2 is associated with poor prognosis in ACC (HR = 1.07, *P* < 0.0001), KICH (HR = 1.35, *P* = 0.027), KIRC (HR = 1.05, *P* = 0.017), KIRP (HR = 1.11, *P* < 0.0001), LGG (HR = 1.03, *P* < 0.0001), LIHC (HR = 1.04, *P* = 0.00027), MESO (HR = 1.08, *P* < 0.0001), PAAD (HR = 1.05, *P* = 0.02), PRAD (HR = 1.11, *P* = 0.0018), SARC (HR = 1.02, *P* = 0.0016), and SKCM (HR = 1.01, *P* = 0.00031). On the contrary, the high expression of GINS2 is associated with the protective factor in THCA and THYM tumours. The correlation analysis between GINS2 expression and DSS, increased GINS2 gene expression in patients with ACC, KICH, KIRC, KIRP, LGG, LIHC, MESO, PAAD, PRAD, SARC, and SKCM tumours, is associated with poor prognosis. However, the prognosis is good for COAD. In the correlation analysis between GINS2 expression and DFI, GINS2 expression affects poor DFI prognosis in LGG, LIHC, SARC, and THCA. In the correlation analysis between GINS2 expression and PFI, the differences of ACC, KICH, KIRC, KIRP, LGG, LIHC, MESO, PAAD, PCPG, PRAD, SARC, and SKCM in PFI prognosis are statistically significant. In patients with LGG, LIHC, and SARC, high expression of GINS2 is correlated with the poor prognosis of OS, DSS, DFI, and PFI. Therefore, GINS2 is an important factor affecting the prognosis of tumours.

### 3.3. Correlation of GINS2 with Tumour Immune Infiltration and Immune Microenvironment

**Figure 4:**
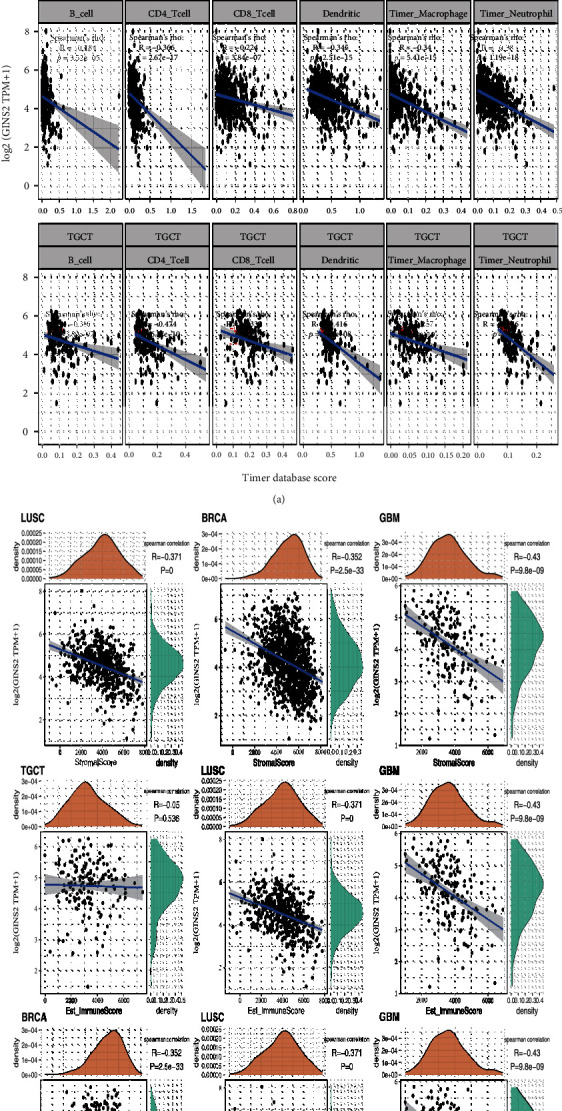
Correlation analysis between GINS2 expression in pan-cancer and tumour immune infiltration and tumour microenvironment. (a) Correlation analysis between expression levels of GINS2 and immune cell infiltration in LGG. Correlation analysis between expression levels of GINS2 and immune cell infiltration in LUSC. Correlation analysis between the expression level of GINS2 and immune cell infiltration in TGCT. (b) Correlation analysis between GINS2 expression in pan-cancer and immune score, GINS2 expression and stromal score, and GINS2 expression and estimate immune score.

Based on the TIMER database, the scores of six kinds of infiltrating immune cells related to 33 tumours (B cells, CD4+ T cells, CD8+ T cells, neutrophils, macrophages, and dendritic cells) were calculated, and the correlation of GINS2 expression with these immune cells was analyzed. The immune infiltration analysis showed that the expression of GINS2 is correlated with the immune infiltration level in different cancers. The results of three tumours with the most significant correlation (LGG, LUSC, and TGCT) are only presented here (Figure 4(a)). In addition, through analyzing the immune score and interstitial score of a single tumour sample, the correlation between GINS2 and the tumour immune microenvironment was analyzed (Figure 4(b)). Amongst the 33 tumour samples, the top three tumours with the most significant correlation between GINS2 and matrix score are LUSC (*R* = −0.371, *P* = 0), BRCA (*R* = −0.352, *P* = 2.5*e* − 33), and GBM (*R* = −0.43, *P* = 9.8*e* − 09). Amongst them, the expression of GINS2 is negatively correlated with the matrix score. The top three tumours with the most significant correlation between GINS2 and Est_ImmuneScore include TGCT (*R* = −0.05, *P* = 0.536), LUSC (*R* = −0.371, *P* = 0) and GBM (*R* = −0.43, *P* = 9.8*e* − 09). Amongst them, the expression of GINS2 is negatively correlated with Est_ImmuneScore. The top three tumours with the most significant correlation between GINS2 and ESTIMATEScore include BRCA (*R* = −0.352, *P* = 2.5*e* − 33), LUSC (*R* = −0.371, *P* = 0), and GBM (*R* = −0.43, *P* = 9.8*e* − 09). Amongst them, the expression of GINS2 is negatively correlated with ESTIMATEScore.

### 3.4. GINS2 Expression Correlated with Immune Neoantigen and Immune Checkpoint Genes

**Figure 5:**
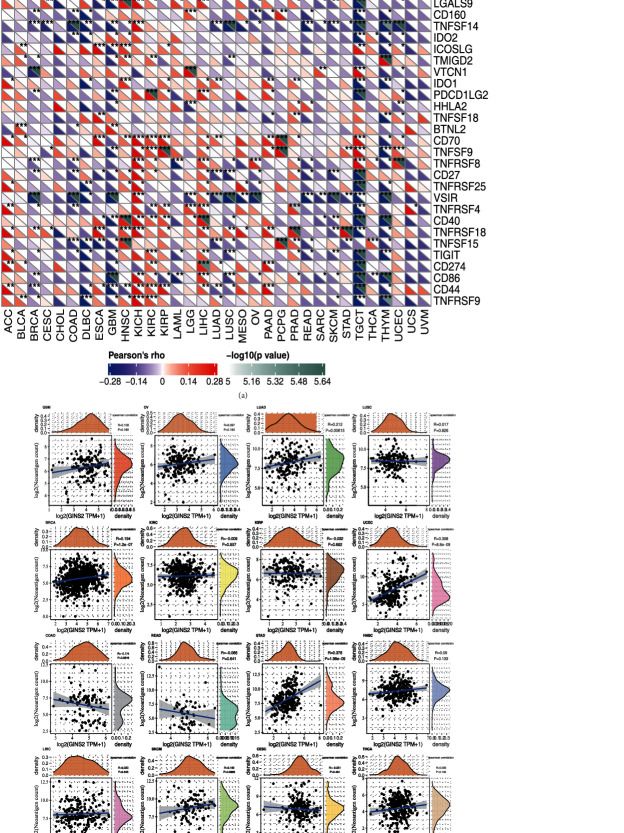
Correlation analysis between GINS2 expression and immune neoantigens and immune checkpoint genes in pan-cancer. (a) Correlation analysis between GINS2 expression in pan-cancer and immune checkpoint gene expression. ^∗^Significant correlation *P* < 0.05. ^∗∗^Significant correlation *P* < 0.01. ^∗∗∗^Significant correlation *P* < 0.001. (b) Correlation analysis between GINS2 expression in pan-cancer and the number of tumour neoantigens in 19 tumours.

Our study findings confirm that the expression of GINS2 in different types of tumours is associated with gene expression in immune checkpoints. In KICH, KIRC, HNSC, and other tumours, the expression of GINS2 is positively correlated with the gene expression in immune checkpoints. In DLBC, GBM, TGCT, THYM, and other tumours, the expression of GINS2 is negatively correlated with gene expression in immune checkpoints. Therefore, GINS2 can regulate the expression of some related immune checkpoint genes in some tumours, to play an essential role in regulating tumour-resistant mode (Figure 5(a)) (^∗^ represents correlation *P* < 0.05, ^∗∗^ represents correlation *P* < 0.01, and ^∗∗∗^ represents correlation *P* < 0.001). The number of neoantigens in each tumour sample was counted, and the relationship between GINS2 expression and the number of these neoantigens was analyzed. The results showed that the expression of GINS2 is positively correlated with the number of neoantigens only in STAD (*R* = 0.376, *P* < 0.001) and UCEC (*R* = 0.358, *P* < 0.001) (Figure 5(b)).

### 3.5. GINS2 Expression Correlated with Tumour Mutation Burden and MSI in Pan-Cancer

**Figure 6:**
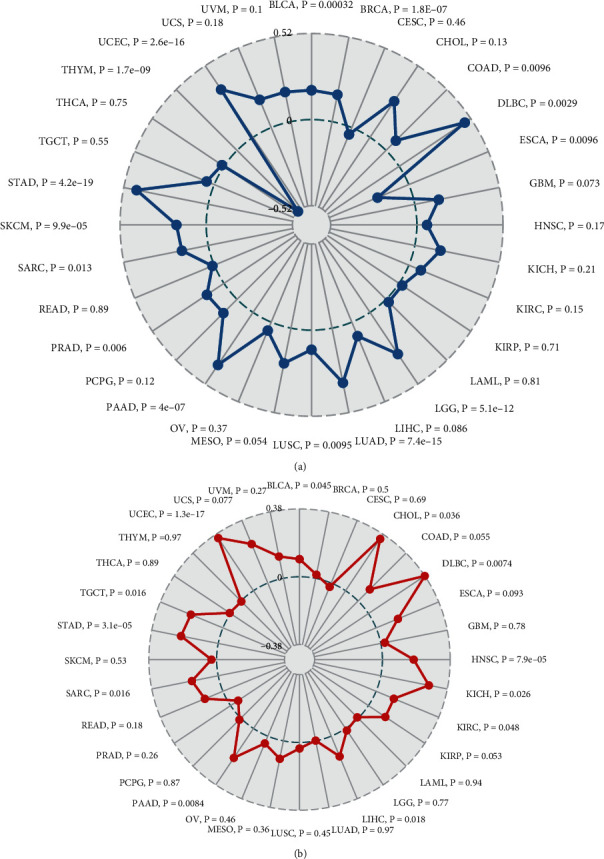
Relation between TMB, MSI, and GINS2 mRNA expression levels in various tumours in TCGA database. (a) Correlation between TMB and GINS2 expression. (b) Correlation between MSI and GINS2 expression. The Spearman correlation test, *P* < 0.05 was considered significant.

The correlation between TMB and GINS2 expression in various tumours was statistically analyzed by the Spearman rank correlation coefficient. In BLCA, BRCA, DLBC, LGG, LUAD, LUSC, PAAD, PRAD, SARC, SKCM, STAD, and UCEC, the expression of GINS2 is positively correlated with TMB and negatively correlated with ESCA and THYM (Figure 6(a)). The correlation between GINS2 expression and MSI was analyzed by using the Spearman rank correlation coefficient. The results showed that in BLCA, CHOC, DLBC, HNSC, KICH, KIRC, LIHC, PAAD, SARC, STAD, TGCT, and UCEC, the expression of GINS2 is positively correlated with MSI, and there is no negative correlation (Figure 6(b)). The above data showed that high expression of GINS2 is extensively associated with cancer immunity.

### 3.6. GINS2 Affected the Expression of DNA MMR Genes and Methyltransferase in Pan-Cancer

**Figure 7:**
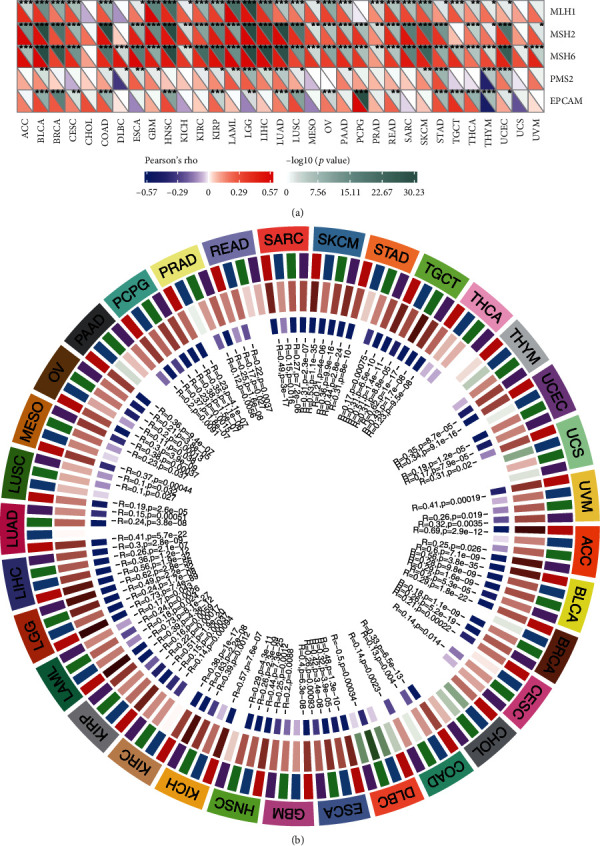
Relationship between MMR defects, methylation levels, and GINS2 mRNA expression level in various tumours in TCGA database. (a) Correlation between GINS2 mRNA expression and mutation levels of five significant MMR genes (MLH1, MSH2, MSH6, PMS2, and EPCAM). The lower triangle in each tile indicates coefficients calculated by Pearson's correlation test, and the upper triangle indicates log10 transformed *P* value. ^∗^*P* < 0.05, ^∗∗^*P* < 0.01, and ^∗∗∗^*P* < 0.001. (b) Correlation between GINS2 and four methyltransferases (DNMT1: red; DNMT2: blue; DNMT3A: green; DNMT3B: purple) mRNA levels.

Except for CHOL and UCS, almost all MMR genes are positively correlated with the expression of GINS2. This finding indicates that GINS2 can maintain the vitality of tumour cells by upregulating DNA MMR-related genes (Figure 7(a)). The relationship between the expression of GINS2 and four methyltransferases was studied through correlation visual analysis. The results confirmed that GINS2 presentation shows a significantly positive correlation with methyltransferase expression in most tumours, especially in BLCA, KICH, KIRP, LAML, LGG, LIHC, and TGCT (Figure 7(b)). This finding indicates that GINS2 can regulate tumour genesis and progression by regulating human epigenetic status.

### 3.7. Correlation between GINS2 and CAFs

**Figure 8:**
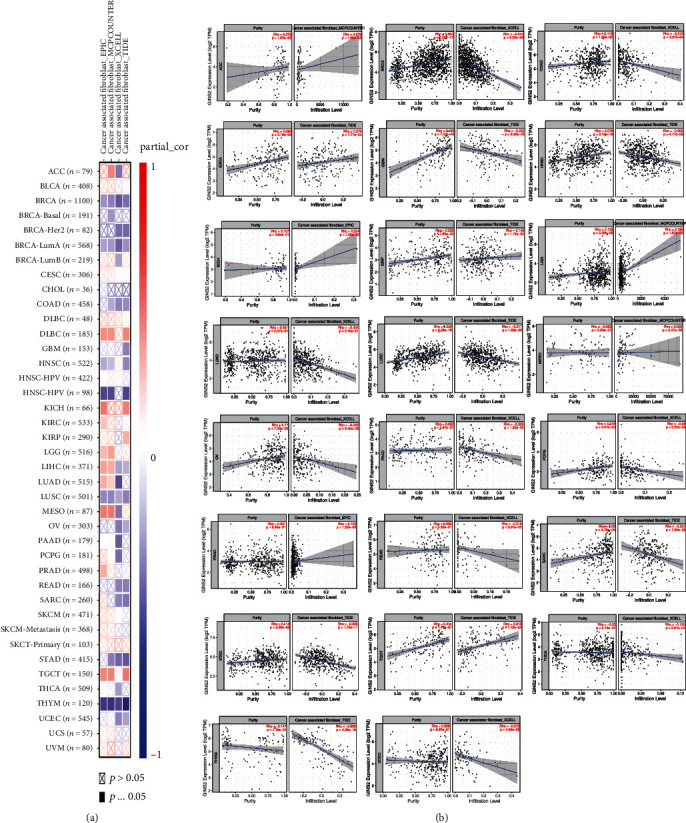
The relationship between GINS2 expression and immune infiltration of cancer-associated fibroblasts (CAFs). (a) Four algorithms (EPIC, MCPCOUNTER, XCELL, and TIDE) were used to investigate the possible relationship between GINS2 expression and infiltration of cancer-associated fibroblasts in various cancer types. (b) Moreover, the results yielded appropriate conclusions.

CAFs are generally considered to have tumourigenic properties. The correlation between GINS2 expression and CAFs was studied based on four algorithms: EPIC, MCPCOUNTER, XCELL, and TIDE. The results showed that the expression of GINS2 is negatively correlated with CAF abundance in most cancer types in BRCA, COAD, GBM, HNSC, LUAD, LUSC, OV, PAAD, PCPG, READ, SARC, STAD, THCA, THYM, and UCEC tumours extracted from TCGA database; the expression of GINS2 is negatively correlated with the infiltration of CAFs. In ACC, ESCA, KICH, KIRP, LGG, MESO, PRAD, and TGCT tumours, the expression of GINS2 positively correlates with CAF infiltration of CAFs. The data of the above scatter diagrams were obtained by using an algorithm. For example, based on the MCPCOUNTER algorithm, the expression of GINS2 in ACC is positively correlated with the infiltration of CAFs (cor = 0.279, *P* = 1.66*e* − 02) (Figures 8(a) and 8(b)).

### 3.8. GSEA Analysis on GINS2

**Figure 9:**
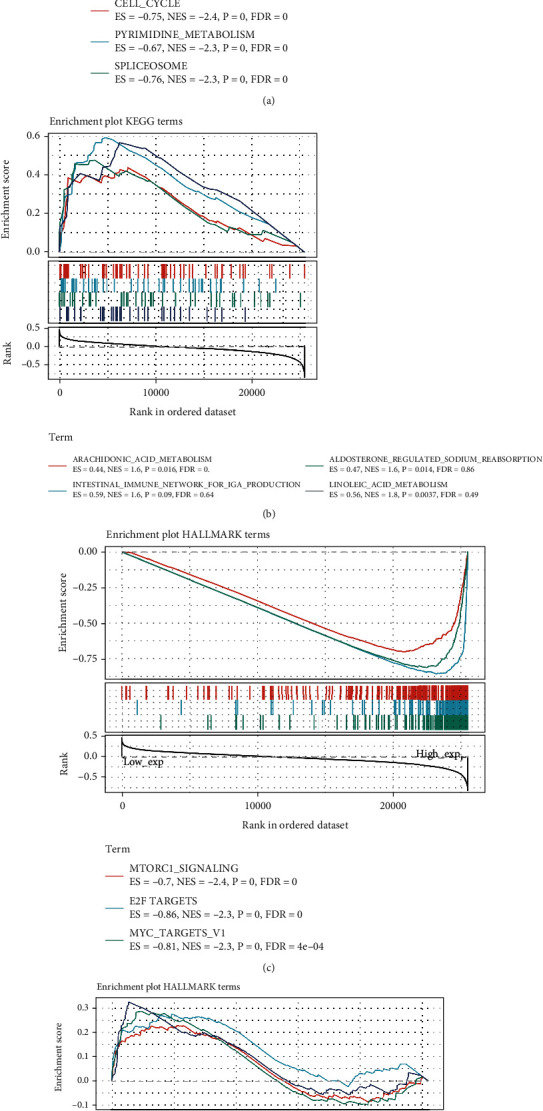
GSEA in the high GINS2 group and low GINS2 group. (a) Enrichment analysis of KEGG pathway in the high GINS2 expression group. (b) Enrichment analysis of KEGG pathway in the low GINS2 expression group. (c) Pathway analysis of HALLMARK by the high GINS2 group. (d) Enrichment in HALLMARK by samples with low GINS2 expression.

GSEA identified the functional enrichment of high and low expressions of GINS2 (Figure 9). KEGG enrichment item shows that high expression of GINS2 is mainly related to tyrosine kinase signaling pathways, including cell cycle pathway, pyrimidine metabolism signaling pathway, and spliceosome pathway. The hallmark item shows that high expression of GINS2 is related to MTORC1 signaling, E2F targets, and MYC target-U1 signaling pathways.

### 3.9. Analysis of GINS2 and Its Related Proteins

**Figure 10:**
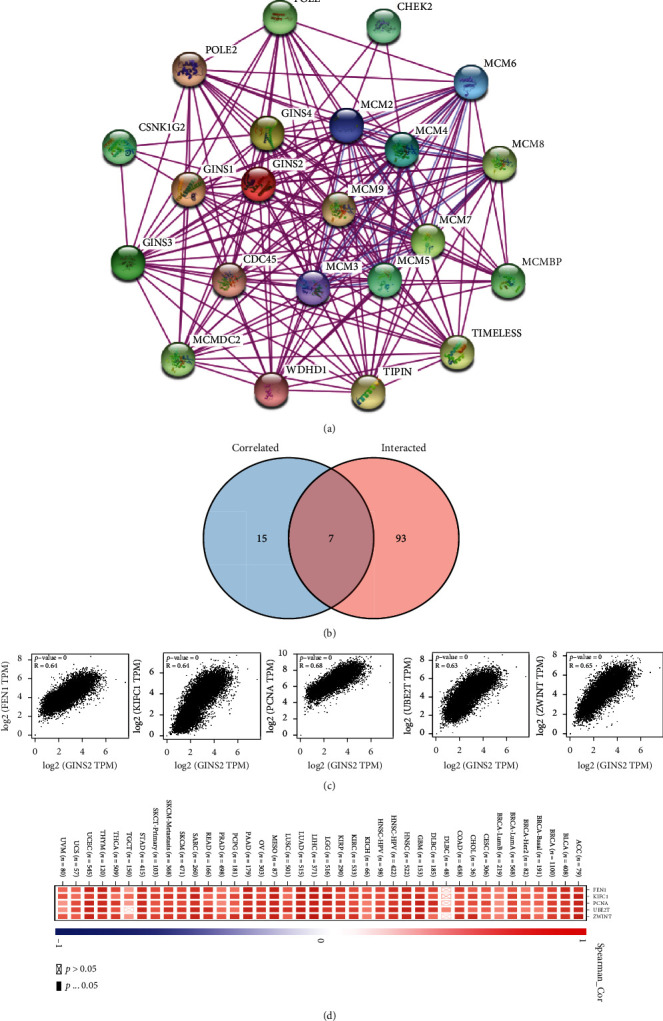
Enrichment analysis of the GINS2 gene. (a) A total of 22 proteins that bind to GINS2 were identified using the STRING tool. (b) In addition, 100 genes associated with GINS2 were acquired from TCGA database, and the data demonstrated the relevance of five genes (GINS1, MCM2, MCM3, MCM6, TIMELESS, POLE2, and CDC45) interacting with GINS2. (c) The association of 5 genes with the incidence of various cancer types was examined. (d) The cross-tabulation of the genes was obtained from these two datasets.

To further explore the potential molecular mechanism of GINS2 in tumourigenesis, we selected the related genes that target the expression of GINS2 and conducted a series of analyses. STRING tool was used to identify 22 binding proteins in the GINS2 gene.  Figure 10(a) shows the interactive network of these genes. Then, all TCGA tumour expression data were evaluated by using the GEPIA2 tool to obtain the top 100 genes related to GINS2 expression. The correlation of GINS2 expression with flap structure-specific endonuclease 1 (FEN1) (*R* = 0.64), kinesin family member C1 (KIFC1) (*R* = 0.64), proliferating cell nuclear antigen (PCNA) (*R* = 0.68), ubiquitin-conjugating enzyme E2T (UBE2T) (*R* = 0.63), and ZW10 interacting kinetochore protein (ZWINT) (*R* = 0.65) (all *P* < 0.001) is shown by the scatter diagram (Figure 10(b)). The corresponding heat maps in most detailed cancer types further prove that GINS2 is positively correlated with the five genes mentioned above (Figure 10(c)). There are seven common members in the above two data sets, namely, GINS complex subunit 1 (GINS1), minichromosome maintenance complex component 2 (MCM2), minichromosome maintenance complex component 3 (MCM3), minichromosome maintenance complex component 6 (MCM6), timeless circadian regulator (TIMELESS), DNA polymerase epsilon 2, accessory subunit (POLE2), and cell division cycle 45 (CDC45) (Figure 10(d)).

## 4. Discussion

As an oncogene, GINS2 can promote cell growth and inhibit cell apoptosis in various cancers [19]. For example, the upregulation of GINS2 can promote the tumour progression of NSCLC, and the knockdown of GINS2 can inhibit the proliferation of human glioma cells [20]. Whether GINS2 can play a role in the pathogenesis of different tumours through some of the exact molecular mechanisms still needs further investigation. Through a literature search, we could not find any published papers on the pan-cancer analysis of GINS2 from the perspective of the tumour as a whole. Therefore, this study provides a relatively comprehensive content of pan-cancer analysis and thoroughly analyzes the role of GINS2 in cancer. The results show that GINS2 overexpression is related to poor prognosis in some types of cancer. GINS2 expression is closely related to the level of immune infiltration. In addition, GINS2 is abnormally expressed in various cancers, which is significantly associated with MMR, MSI, DNA methylation, and TMB. Therefore, GINS2 may play a crucial role in cancer prognosis and tumour immunity [21].

Firstly, our results show that compared with nontumour tissues, the high expression of GINS2 in BLCA, BRCA, CHOL, COAD, ESCA, GBM, HNSC, KICH, KIRC, KIRP, LIHC, LUAD, LUSC, STAD, THCA, UCEC, CESC, READ, PCPG, ACC, DLBC, LAML, OV, SARC, SKCM, THYM, and UCS tissues indicates that GINS2 may be used as a tumour promoter in human cancer [22]. This is consistent with previous findings [20, 23, 24]. Secondly, combined with the results of the Cox analysis and Kaplan–Meier method, the high expression of GINS2 is associated with poor OS rate in ACC, KICH, KIRC, KIRP, LGG, LIHC, MESO, PAAD, PRAD, SARC, and SKCM and good OS rate in THCA and THYM. In addition, the increase in GINS2 expression in ACC, KICH, KIRC, KIRP, LGG, LIHC, MESO, PAAD, PRAD, SARC, and SKCM indicates poor DSS but good DSS in COAD. In LGG, LIHC, SARC, and THCA, the increase in GINS2 expression indicates poor DFI. In ACC, KICH, KIRC, KIRP, LGG, LIHC, MESO, PAAD, PCPG, PRAD, SARC, and SKCM, the increase in GINS2 expression shows poor PFI. Based on our results, GINS2 can be used as a prognostic biomarker for some malignant tumours [25]. The high expression of GINS2 may promote the death of cancer patients. The clinical significance of GINS2 in pan-cancer was studied. The expression of GINS2 is higher in ACC, HNSC, KIRC, KIRP, LUAD, and TGCT and lower in KICH and LIHC. These results suggest that GINS2 may be an essential player in the development of tumour progression. As a pivotal component of the tumour microenvironment, tumour immune infiltrating cells play a potential regulatory role in the advancement of various tumours [26]. Interestingly, the expression of GINS2 is significantly correlated with multiple immune infiltration levels in human cancer, especially in LGG, LUSC, and TGCT, which indicates that GINS2 may mediate cancer progression by affecting immune infiltration in malignant tumours. TMB and MSI are common in human cancer and can be used as predictors of cancer treatment efficacy [27].

The expression of GINS2 in BLCA, BRCA, DLBC, LGG, LUAD, LUSC, PAAD, PRAD, SARC, SKCM, STAD, and UCEC is positively correlated with TMB and negatively correlated with ESCA and THYM. In addition, in BLCA, CHOC, DLBC, HNSC, KICH, KIRC, LIHC, PAAD, SARC, STAD, TGCT, and UCEC, the high expression of GINS2 shows a significantly positive correlation with MSI. It is worth noting that in BLCA DLBC, PAAD, SARC, SARC STAD, and UCEC several kinds of cancer, GINS2 expression is positively related with TMB with MSI, suggesting that these types of cancer patients with high expression of GINS2 checkpoint inhibitors to vaccination may have a better response. Methyltransferase is an epigenetic feature with good characteristics in malignant tumours. Some methyltransferases have been verified as therapeutic targets [28, 29]. Moreover, tumours with MMR protein defects may be more susceptible to immune checkpoint blockade [28, 30]. This study analyzed the correlation between GINS2 and methyltransferase and MMR proteins. The results showed that the expression of GINS2 is significantly correlated with methyltransferases and MMR proteins in various tumours, indicating the critical relationship between GINS2 and tumour immunity.

However, this study has some limitations, which cannot be ignored [31]. Firstly, we comprehensively evaluated the mRNA levels of GINS2. The evidence for its correlation with protein levels in human cancer is insufficient [32]. Secondly, the sample size was small. It is necessary to extract a larger sample size from other public data sets to verify further and supplement our current findings [28]. Finally, multiple pieces of information were retrieved from different databases for analysis, so there is a specific systematic deviation [33]. Therefore, we must make more efforts to explore the effects of GINS2 in cancer and the value of GINS2 as a potential target in anticancer therapy.

## 5. Conclusions

This study shows that GINS2 is related to the prognosis of cancer patients and the immune infiltration of different cancers. The expression of GINS2 is associated with MMR, MSI, TMB, and DNA methylation in various cancers. The expression of GINS2 is closely related to the expression of immune genes in various cancers. GINS2 may play a vital role as a prognostic biomarker.

## Data Availability

The data that we used to support the findings were mentioned in this study.

## Supplementary Materials

Supplementary Materials We explored the correlation between GINS2 gene expression and tumour survival and prognosis in TCGA and gave the Kaplan-Meier curves of positive DSS, DFI, and PFI results of different tumours in the supplementary materials. Supplementary Figure 1: correlation between GINS2 gene expression and survival prognosis of cancers in TCGA. The Kaplan–Meier curves of DSS in different tumours with positive results are offered. Supplementary Figure 2: correlation between GINS2 gene expression and survival prognosis of cancers in TCGA. The Kaplan–Meier curves of DFI in different tumours with positive results are offered. Supplementary Figure 3: correlation between GINS2 gene expression and survival prognosis of cancers in TCGA. The Kaplan–Meier curves of PFI in different tumours with positive results are offered.
