# Supplementary figures and images for: Comprehensive Pan-Cancer Analysis of GINS2 for Human Tumour Prognosis and as an Immunological Biomarker
Source: Comput Math Methods Med. 2022 Nov 23;2022:3119721. doi: 10.1155/2022/3119721 (PMC9711967; doi:10.1155/2022/3119721)

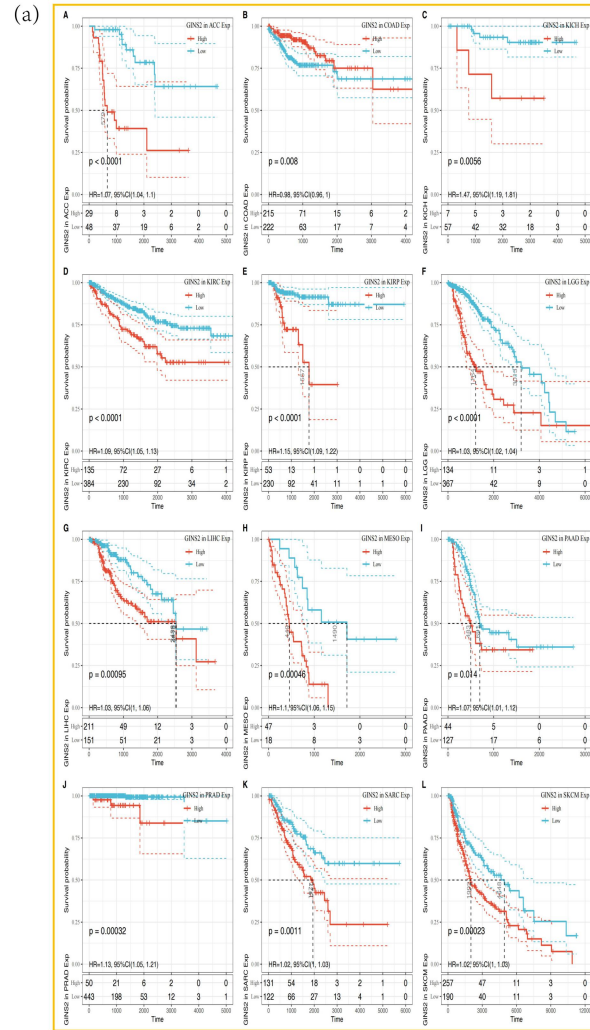

Supplementary  
Figure 1

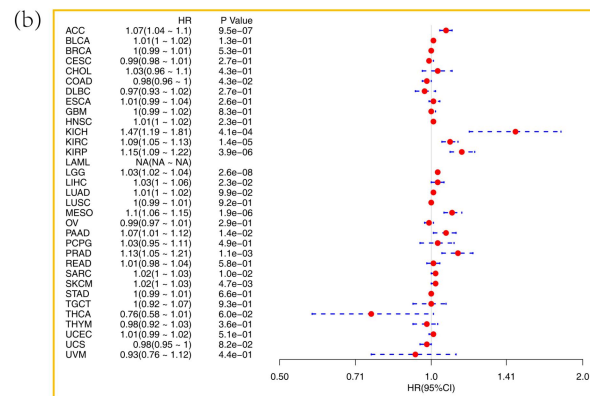

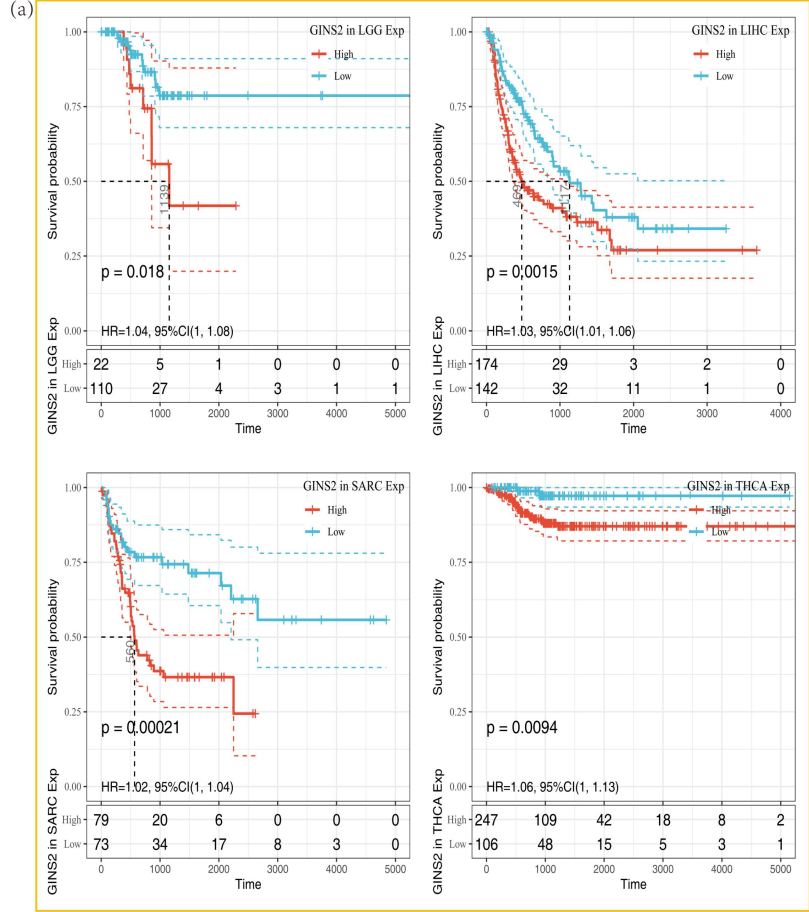

Supplementary  
Figure 2

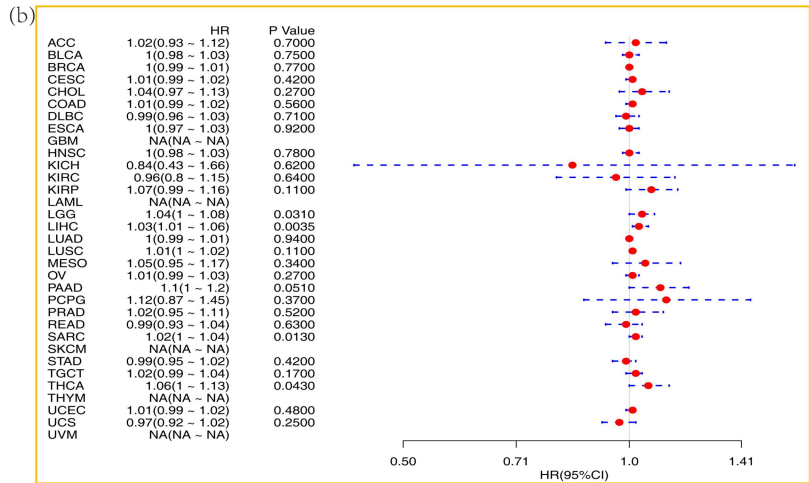

(a)

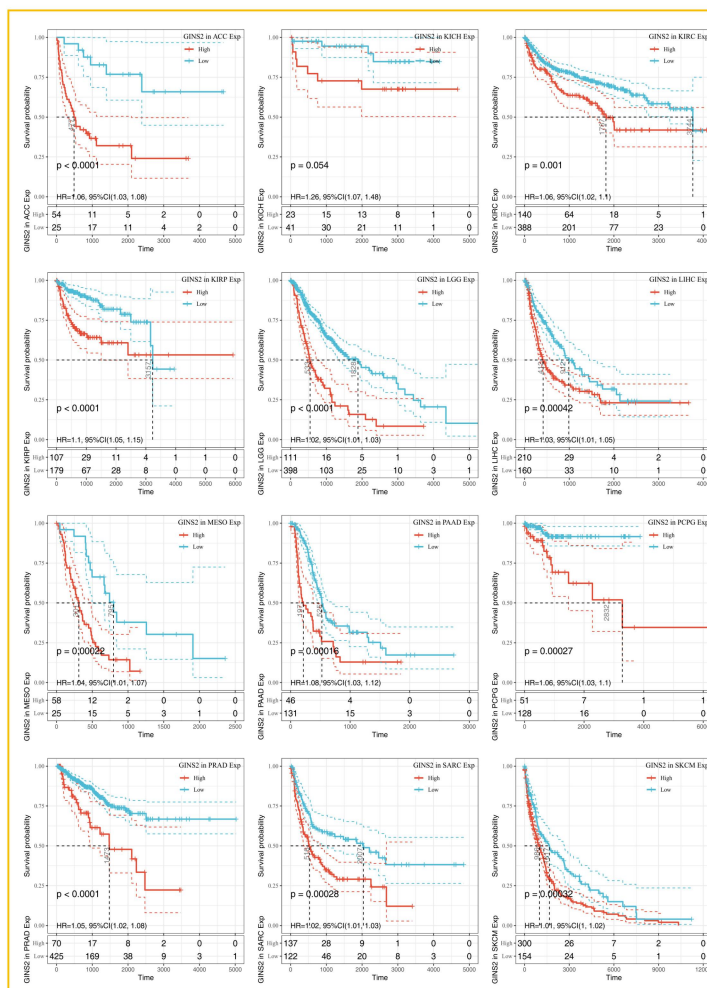

Supplementary  
Figure 3

(b)

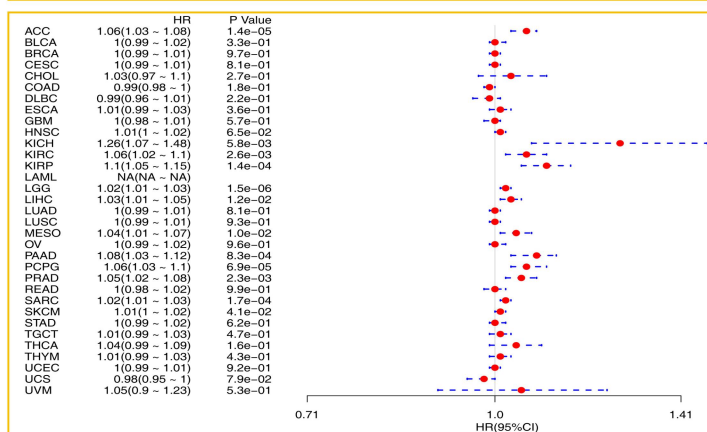

Supplement: Supplementary Materials — We explored the correlation between GINS2 gene expression and tumour survival and prognosis in TCGA and gave the Kaplan-Meier curves of positive DSS, DFI, and PFI results of different tumours in the supplementary materials. Supplementary Figure 1: correlation between GINS2 gene expression and survival prognosis of cancers in TCGA. The Kaplan–Meier curves of DSS in different tumours with positive results are offered. Supplementary Figure 2: correlation between GINS2 gene expression and survival prognosis of cancers in TCGA. The Kaplan–Meier curves of DFI in different tumours with positive results are offered. Supplementary Figure 3: correlation between GINS2 gene expression and survival prognosis of cancers in TCGA. The Kaplan–Meier curves of PFI in different tumours with positive results are offered. [file 3119721.f1.pdf]
